# Quality of Psychoeducational Apps for Military Members With Mild Traumatic Brain Injury: An Evaluation Utilizing the Mobile Application Rating Scale

**DOI:** 10.2196/19807

**Published:** 2020-08-18

**Authors:** Chelsea Jones, Kaitlin O'Toole, Kevin Jones, Suzette Brémault-Phillips

**Affiliations:** 1 Heroes in Mind, Advocacy and Research Consortium (HiMARC) Faculty of Rehabilitation Medicine University of Alberta Edmonton, AB Canada; 2 Canadian Forces Health Services Department of National Defence Government of Canada Ottawa, ON Canada; 3 Edmonton North Primary Care Network Edmonton, AB Canada

**Keywords:** psychoeducation, mTBI, military, app, smartphone, mHealth, concussion, Mobile App Rating Scale, MARS, mobile phone

## Abstract

**Background:**

Military personnel have an elevated risk of sustaining mild traumatic brain injuries (mTBI) and postconcussion symptoms (PCS). Smartphone apps that provide psychoeducation may assist those with mTBI or PCS to overcome unique barriers that military personnel experience with stigma and access to health care resources.

**Objective:**

This study aims to (1) use the Mobile Application Rating Scale (MARS) to evaluate smartphone apps purporting to provide psychoeducation for those who have sustained an mTBI or a PCS; (2) explore the relevance, utility, and effectiveness of these apps in facilitating symptom management and overall recovery from mTBI and PCS among military personnel; and (3) discuss considerations pertinent to health care professionals and patients with mTBI when considering the use of mobile health (mHealth), including apps for mTBI psychoeducation.

**Methods:**

A five-step systematic search for smartphone apps for military members with mTBI or PCS was conducted on January 31, 2020. Cost-free apps meeting the inclusion criteria were evaluated using the MARS and compared with evidence-based best practice management protocols for mTBI and PCS.

**Results:**

The search yielded a total of 347 smartphone apps. After applying the inclusion and exclusion criteria, 13 apps were subjected to evaluation. Two apps were endorsed by the US Department of Veterans Affairs and the US Department of Defense; all the others (n=11) were developed for civilians. When compared with evidence-based best practice resources, the apps provided various levels of psychoeducational content. There are multiple considerations that health care professionals and those who sustain an mTBI or a PCS have to consider when choosing to use mHealth and selecting a specific app for mTBI psychoeducation. These may include factors such as the app platform, developer, internet requirement, cost, frequency of updates, language, additional features, acknowledgment of mental health, accessibility, military specificity, and privacy and security of data.

**Conclusions:**

Psychoeducational interventions have a good evidence base as a treatment for mTBI and PCS. The use of apps for this purpose may be clinically effective, cost-effective, confidential, user friendly, and accessible. However, more research is needed to explore the effectiveness, usability, safety, security, and accessibility of apps designed for mTBI management.

## Introduction

### Background

Mobile health (mHealth) is an emerging field, with health care professionals increasingly using apps as part of clinical practice [1]. Apps geared toward well-being and health promotion have drastically increased in number and availability and may offer new opportunities for individuals seeking immediate and confidential health care [2,3]. One population that has the potential to benefit from the use of such apps is public safety personnel and military members [4]. Canadian Armed Forces (CAF) service members (SMs), similar to other global militaries, have significant mental and physical health challenges associated with unique occupational stressors associated with military duties [4,5]. Military service commonly involves high-risk activities during physical training, daily trade-related tasks, overseas deployment, and responses to natural disasters among others. Their involvement in such activities can increase their likelihood of sustaining physical and mental health injuries, including mild traumatic brain injury (mTBI) [5,6].

#### mTBI

mTBI, also known as concussion, is defined as a temporary change in brain functioning caused by an insult to the head with a period of posttraumatic amnesia lasting less than a day [7,8]. In contrast, moderate and severe TBI include changes in brain functioning resulting from a head insult causing periods of posttraumatic amnesia lasting longer than a day and often a period of hospitalization in an acute care facility and/or tertiary rehabilitation [7,8]. Symptoms of mTBI may include headaches, fatigue, nausea, sensitivity to light and sound, visual disturbances, cognitive dysfunction, memory loss, sleep disturbances, balance or vestibular issues, emotional disturbances, seizures, and loss of consciousness to name a few [7-20]. Symptoms of mTBI generally resolve within 2 weeks when no additional physical or mental comorbidities and extenuating factors are present [7,8]. If 3 or more symptoms of mTBI persist for longer than 3 months, a diagnosis of postconcussion symptoms (PCS) may be made [5,7,8].

#### Incidence of mTBI and PCS Among Military Populations

The cause of an mTBI varies among CAF-SMs, with some occurring as a result of motor vehicle collisions, falls, sports, explosions, or other forces related to combat and military training [9-16,18]. Rates of mTBI prevalence and severity of symptoms vary by element (ie, Army, Navy, or Air Force), age, gender, trade or profession, and unit [18]. Military members who experience an mTBI in combat may be at risk of developing career-limiting medical conditions [16]. As of 2019, mTBI affected 1 in 25 CAF-SMs, with 5.7% female and 3.9% male CAF-SMs diagnosed with mTBI over a 5-year period (2012-2017) [18]. Notably, this was after the completion of CAF’s involvement in Operation Iraqi Freedom (OIF) and Operation Enduring Freedom (OEF), indicating that these incidences of mTBI took place largely outside of combat zones [18]. Among CAF-SMs deployed to Afghanistan during OEF between 2009 and 2012, 5.2% self-reported experiencing an mTBI, 21% of whom noted PCS [5,7]. In comparison, US studies among military populations report mTBI rates of 12% to 22.8% during OEF and OIF, with PCS rates of 15.8% to 35% [9-11,14]. The UK Armed Forces report a 4.4% mTBI prevalence among SMs deployed into these global conflicts [11]. Although numbers vary greatly between different global militaries, the evidence base consistently demonstrates higher rates of mTBI and PCS in military personnel than in civilian populations. Similarly, although the incidence of PCS among the global civilian population has been estimated at 15% [8], it is well documented that this rate is elevated among military populations, with global estimates ranging from 15.8% to 35% [5,9,12,14-21]. This is because of a host of factors that are more prevalent among military populations than civilians, including a higher incidence of mental health disorders, exposure to traumatic experiences, previous mTBI, stigma, and a general lack of knowledge about mTBI [9,12,14-16].

Military members experience a higher incidence of posttraumatic stress disorder (PTSD), anxiety, and depression, which can have significant functional implications when co-occurring with mTBI. mTBI or TBI and mental health disorders, such as PTSD, can co-occur from the same or separate traumatic incidents [6,7,9-16]. The presence of trauma, as well as previously diagnosed neurological or mental health disorders, has been demonstrated to exacerbate mTBI symptoms and may be a major factor in the presence, longevity, and severity of PCS [5,12,13]. Additionally, it is possible that military members have had multiple previous mTBI that may or may not have been formally diagnosed [18]. The compounding effects of subsequent mTBI have been researched in recent years; however, the severity, longevity, and specificity of these symptoms and subsequent dysfunction they may cause are still widely unknown [7,8].

In addition to symptoms and stressors directly attributed to mTBI, psychosocial stressors may also be experienced by military members. Such stressors may include social and geographical isolation as well as concerns regarding medical employment limitations. It is widely acknowledged that mTBI is underreported both in the CAF and other global militaries because of several factors including stigma, fear of career implications, and ignorance of the potential seriousness of mTBI [9,12,18]. Seeking medical care and receiving an mTBI diagnosis may result in time away from work or absences from courses, training exercises, or deployments [13,16,18]. Military members may not have awareness that resources and interventions available through primary care, physical rehabilitation, and mental health could assist with recovery from mTBI or PCS. Widespread education about mTBI and treatment options that reduce perceived or actual stigma and threats to careers may be effective in reducing the negative impact of mTBI and PCS.

#### Psychoeducation for mTBI Among Military Populations

Various interventions for treating mTBI symptoms have been studied among military populations [15-19,21]. A 2015 study reviewing the effectiveness of interventions for military members with mTBI, PCS, and mental health comorbidities isolated 4 categories of interventions: psychoeducation, psychotherapy, cognitive rehabilitation therapy, and integrated behavioral health interventions [15]. Psychoeducational interventions have a strong evidence base both as treatments for mTBI and as supplements to therapies for mental health disorders in both the acute and chronic phases of the illness [8,14,15,17,19-21]. Access to appropriate and timely psychoeducation is important to facilitate timely recovery from mTBI [8,15,17,19-21]. Providing psychoeducation directly after sustaining a mTBI and during the chronic phase (ie, PCS) has been demonstrated to reduce the impact and longevity of somatic symptoms and the potential exacerbation of mental health distress [8,15,17-20].

Military members require psychoeducational interventions that are clinically effective, cost-effective, user friendly, available in multiple environments, secure, and confidential [3]. This is particularly important when in-person therapy is not possible, such as during deployment, natural disaster response, or a pandemic. At such times, it is essential that clinicians explore more novel interventions and modes of service delivery, which may include the use of smartphone apps. As apps have evolved with better accessibility, usability, and quality, the delivery of psychoeducational material for behavioral change and health improvement via this method has become more common. This paper will refer to *apps* as opposed to *applications* in accordance with recommendations Lewis et al [22] in 2014.

#### mHealth and mTBI

As with the civilian population, the use of health apps is becoming more widespread within military populations [3,23]. A 2018 scoping review of mental health mobile apps for use by military members reviewed the current literature aimed at determining whether or not mobile apps are perceived as an acceptable form of mental health support [23]. Studies included in the review addressed app utilization through an assessment of users’ general attitudes of the app, perceived ease of use, and whether they would recommend the app to others [23]. Although the majority of the studies were conducted with the US military, the results of the review overwhelmingly indicated that military members were generally willing to use apps [23] and viewed mobile apps as being an ideal supplement to traditional health care [3,23].

Since the first appearance of an mTBI-based app in 2009, apps specific to mTBI have been rapidly produced and evolving [24]. A 2018 review searching a wide variety of available mTBI-focused apps found 5 general categories: (1) education and prevention, (2) diagnostic assessment, (3) head impact sensors, (4) symptom tracking, and (5) treatment [25]. The most common type of available mTBI apps, and arguably the most controversial in the evidence-based literature, are those in the diagnostic assessment category, which are designed and/or marketed to sports medicine professionals or the general public for use in the recognition and assessment of concussion [24]. Although diagnostic assessment apps may widen the opportunity to identify mTBI, they also provide the potential for less-qualified individuals to use such apps inappropriately [24]. To date, only a limited number of medical devices have been approved or cleared by the Food and Drug Administration to aid in the diagnosis, treatment, or management of head injury, and these do not yet include smartphone apps [26]. Research on apps specific to mTBI has largely focused on the diagnostic abilities of apps and not on the quality of psychoeducational content that these apps provide. This gap in the literature relates especially to the military medical context.

There are both potential benefits and challenges associated with using apps for health and behavioral change. Benefits include decreased stigma and improved privacy, immediate access to psychoeducational content, reduced wait times to access resources, less administrative burden for appointment scheduling, and the ability to track symptoms and share information with health care providers [2,3,27]. There is also evidence to suggest that health care apps may increase help-seeking behaviors and engagement with health care services [27]. Specific to military personnel, the use of mobile technologies for mental health support and care may be a desirable option for many military members and veterans who fear stigmatization and the career implications of engaging with health care systems [23]. Along with the potential benefits of health care apps are potential challenges. Some apps may have a limited evidence base or health care professional involvement during their development phase. This is particularly problematic given that apps in development are not required to undergo any certification or regulatory process, and as many health apps do not utilize peer-reviewed research, their purported claims may not be backed by evidence and may be misleading [2,3,28,29]. Although multiple apps are available specifically for TBI, few guidelines exist to assist individuals in evaluating whether the value of such an app is supported by evidence [24,25].

#### Purpose

The purpose of this evidence-based app review was to (1) use the Mobile Application Rating Scale (MARS) [2] to evaluate smartphone apps that advertise the provision of psychoeducational support for those who have sustained an mTBI or a PCS, (2) explore the relevance, utility, and effectiveness of these apps to facilitate symptom management and overall recovery from mTBI among military personnel, and (3) discuss considerations pertinent to health care professionals and patients with mTBI when considering the use of mHealth, including apps for mTBI psychoeducation. It is hypothesized that multiple high-quality apps exist specific to mTBI in the military population that provide evidence-based, population-specific psychoeducation for mTBI.

## Methods

**Figure 1:**
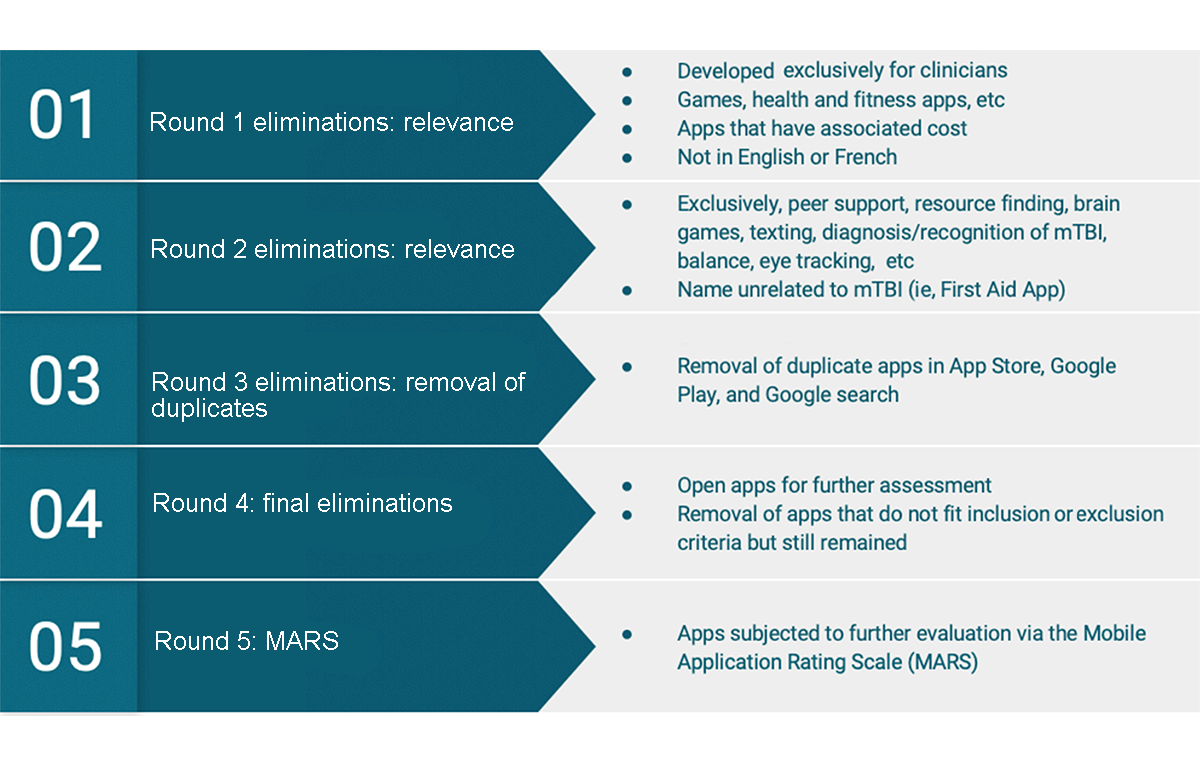
Summary of the 5 rounds of elimination to analyze the apps. mTBI: mild traumatic brain injury.

An app search was conducted on January 31, 2020. The Google Search Engine, the Google Play Store (Canadian) and the Apple App Store (Canadian) were the 3 platforms used for the search. Initial search terms employed with the Google search engine included “military” and “mtbi” or “mild traumatic brain injury” or “concussion” and “apps” or “applications” or “mobile device applications.” Google was selected as the database because of its familiarity, popularity as a search tool, and accessibility from CAF computers. A Google search also provides the user with peer recommendations, which can provide more information about the usefulness of the app for specific populations and may describe features in more detail than the description provided by the 2 app stores. Searches were then conducted on the Apple App Store and the Google Play Store using the terms “concussion” and “mTBI.” The Department of Defense Mobile Health Practice Guide (3rd ed) was also utilized as a starting point; however, it had not been recently updated and did not yield any apps that were not found via the Google search [3]. Review of the apps involved 5 rounds of elimination (Figure 1) and the evaluation of the selected apps using the MARS.

### Rounds of Elimination

In Round 1, the writer screened descriptions from the app stores and excluded apps that were irrelevant, which included games and health and fitness apps unrelated to mTBI. Apps were also excluded if they had an associated cost, were specifically created for use by health care clinicians, were not in English or French, and were not accessible by the author on either the Apple App Store or the Google Play store. In Round 2, apps that were meant for peer support purposes, brain games, text messaging apps, for individuals aged 18 years or younger, and/or specific to 1 symptom of mTBI were also excluded. These included eye-tracking apps, balance apps, and apps aiming to diagnose an mTBI (such as sport-specific sideline apps) that did not have an associated psychoeducational component. Apps were excluded if they did not specifically address mTBI or concussion in the description or title and/or were designed for other purposes (ie, first aid apps). This was because of the assumption that if an app is not specifically designated for concussions or mTBI and does not list this in the name or description, it is unlikely that a person would recognize and choose the app. In Round 3, the remaining apps were compared from the Apple App Store with the Google Play Store to remove duplicates. In elimination Round 4, the writer opened the apps using an iPhone 8 iOS 12.3 (Apple Inc) and Google Pixel XL with Android 9.0. Apps were then excluded if they were intended to be used alongside an in-person therapist or sports coach, or if they otherwise did not include a psychoeducational component for a patient recovering from an mTBI. If the app remained and its purpose was for mTBI detection but it had an educational component, it was included for further consideration. Round 5 involved the use of the MARS for evaluation. A literature search of the 13 apps that remained at Round 5 was conducted using the Scopus, Medical Literature Analysis and Retrieval System Online (MEDLINE), Excerpta Medica dataBASE (EMBASE), and Cumulative Index of Nursing and Allied Health Literature (CINAHL) Plus databases. The aim of this literature search was to determine whether studies using the apps had been published in the evidence-based literature.

### MARS

Once the remaining 13 apps were screened and selected for inclusion in this review, they were evaluated by 3 raters using the MARS [2]. The MARS was created by Stoyanov et al [2] in 2015 for developing an mHealth app rating tool that was reliable, multidimensional, and which could provide a framework to trial, classify, and rate apps. Stoyanov et al [2] developed the MARS and found a high level of interrater reliability for overall MARS scores after evaluating 50 health and well-being apps. The components of the MARS were selected following a literature review to determine existing websites and app evaluation tools and after consulting a multidisciplinary advisory team that included psychologists and mHealth developers [2]. The resulting 3 MARS categories, app quality, app subjective quality, and app specific, were chosen to merge existing mHealth evaluation criteria into a format that was not overly technical, difficult to use, or specific to any one health domain [2].

The app quality category is broken down into 4 subsections: (A) engagement, (B) functionality, (C) aesthetics, and (D) information (Table 1) [2]. Each subsection is averaged with a mean score out of 5. Engagement (A) is addressed using 5 questions and explores the ability of the apps to hold a user’s attention by rating (1) how interesting it was to use, (2) how well-tailored it was to the targeted users, (3) how fun or entertaining it was, and (4) how well it could be customized for each user. Functionality (B) has 4 questions that rate how well the app works for the user. This includes how intuitive the gestures and icons are, how well the components worked when trialed, and how easy it is for a new user to learn. Aesthetics (C) is the shortest subsection, with 3 questions addressing the quality of the visual appeal and graphics. Information (D) is the last and largest subsection, with 7 questions exploring the credibility of the developer and content in the app and whether the app’s efficacy has been tested and reported in published research. This section also addresses how the information within the app is presented to users (eg, is the information accessible to the target audience?) and if the information is of sound quality [2].

App-specific items are available to assess how effectively the app is perceived to address or impact a targeted health behavior [2]. In this study, the targeted health behavior was mTBI management. The 6 app-specific items indicated the rater’s belief that the assessed app would be able to increase the knowledge of, attitude toward, and intention to change health management strategies of someone with an mTBI. The app-specific items also address how strongly the rater felt that the use of the app would actually result in a change in this targeted health behavior. These items were rated on a scale from 1 (strongly disagree) to 5 (strongly agree).

Use of the MARS to rate apps has numerous benefits. Its consideration of a health app based both on design elements (ie, color, graphic resolution, and layout) and content is essential for evaluating quality [2]. Newly published research exploring the quality of apps intended to equip individuals to self-manage their disease reports that apps that have been tested and rated highly on the MARS tool are trustworthy for clinicians to recommend to clients [30]. The MARS was selected to facilitate evaluation of apps in this research project for these features and because it is more comprehensive than the subjective star ratings featured on app stores [4] and is customizable for this specific research focus [2].

Three raters were trained in the MARS by watching the *MARS training video* posted by Stoyanov on YouTube [31]. These raters included a PhD candidate and a CAF Occupational Therapist, an MSc Occupational Therapist, and an American College of Sports Medicine Certified Clinical Exercise Physiologist. The finalized app selection was evaluated by the writers using the MARS January 30, 2020, with apps downloaded onto a Samsung Galaxy Tab A SM-T350 with Android version 7.1.1 software, Google Pixel XL with Android 9.0 software, and/or iPhone 8 iOS 12.3. The results from the raters were averaged to create the final MARS score. The Ontario Neurotrauma Foundations’ Guideline for Concussion/Mild Traumatic Brain Injury and Persistent Symptoms: 3rd Edition, 5th International Conference on Concussion in Sport Concussion Consensus Statement and relevant military-specific evidence-based publications [15,18-20] were used to evaluate the quality and accuracy of the information (Information: Section D of MARS) provided within each app [7,8].

**Table 1:** The Mobile Application Rating Scale.

Sections and subsections		Question number and headings	
**App quality ratings**			
	A. Engagement		Entertainment Interest Customization Interactivity Target group
	B. Functionality		Performance Ease of use Navigation Gestural design
	C. Aesthetics		Layout Graphics Visual appeal
	D. Information		Accuracy of app description Goals Quality of information Quantity of information Visual information Credibility Evidence base
**App subjective quality**			
	N/A^a^		Would you recommend this app to people who might benefit from it? How many times do you think you would use this app in the next 12 months if it was relevant to you? Would you pay for this app? What is your overall star rating of the app?
**App specific**			
	N/A		Awareness Knowledge Attitudes Intention to change Help seeking Behavior change

^a^N/A: not applicable.

## Results

**Figure 2:**
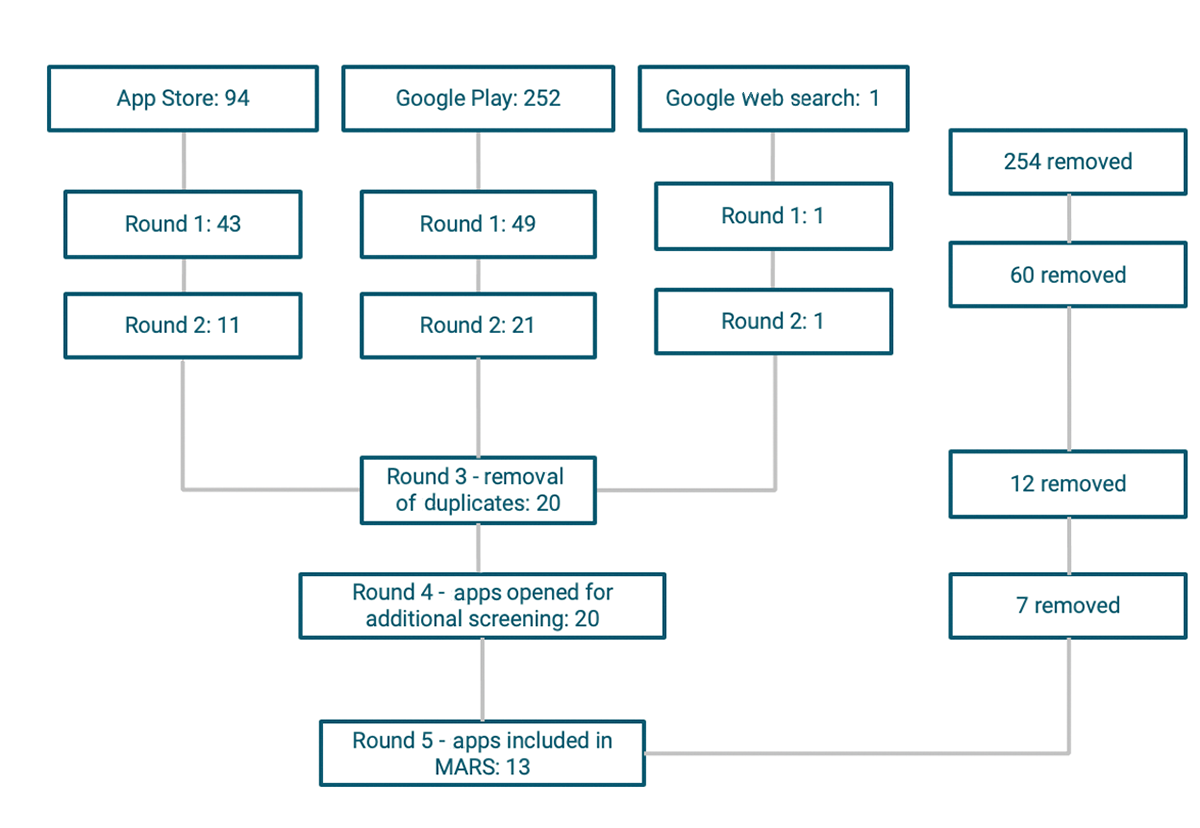
App search results. MARS: Mobile Application Rating Scale.

The search yielded 347 apps that were subjected to 5 rounds of elimination (Figures 1 and 2). The results of the Apple App Store and Google Play Store searches conducted on January 31, 2020, resulted in 13 apps (refer to Figure 2 for a flowchart of the exclusion process). When the Round 1 exclusion criteria were applied, 254 apps from the 347 original apps were excluded as they did not meet the inclusion criteria regarding language, target audience, cost, or relevance. In Round 2, 60 apps were further eliminated because of the goal of the app not specifically targeting psychoeducation for individuals with mTBI. A total of 12 duplicate apps were removed in Round 3. After 20 apps were opened for additional screening, 7 apps were removed in Round 4. This resulted in a total of 13 apps that were evaluated by the writer using the MARS. Information about each of the 13 apps is available in Tables 2 and 3.

Scores for each individual section of the MARS are listed in Table 3. Scores ranged from 1 to 5 out of 5, with higher scores indicating higher engagement, function, information, and overall quality. The interrater reliability was 90% among the raters in all sections of the MARS.

**Table 2:** Individual features of the 13 apps.

App names	Developers	Availability	Log-in	Internet	mTBI^a^ Ax.	Mindfulness	CBT^b^	Goal setting	Symptom tracking	Return to activity	French	Military
Concussion Coach	US Department of Veterans Affairs	Google Play and Apple App Store	No	No	Yes	Yes	Yes	Yes	Yes	No	No	Yes
World Rugby Concussion App	Mobanode	Google Play and Apple App Store	No	No	Yes	No	No	No	Yes	Yes	No	No
CDC Heads Up! Concussion and Helmet Safety App	Centers for Disease Control (CDC)	Google Play and Apple App Store	No	No	No	No	No	No	No	Yes	No	No
Concussion Awareness	Hockey Canada	Google Play and Apple App Store	No	Yes	No	No	No	No	No	Yes	No	No
Concussion Ed	Parachute	Google Play and Apple App Store	No	No	Yes	No	No	No	Yes	Yes	Yes	No
LifeArmor	National Centre for Telehealth and Technology	Google Play and Apple App Store	Yes	No	Yes	Yes	Yes	Yes	Yes	No	No	Yes
CCMI Concussion Tracker	CCM Inc	Google Play and Apple App Store	Yes	Yes	Yes	No	No	No	Yes	No	No	No
Concussion Smart	ABI Ireland/Medtronic	Apple App Store	No	No	Yes	No	No	No	Yes	Yes	No	No
Concussion NI	Sport Northern Ireland	Google Play	No	Yes	No	No	No	No	No	Yes	No	No
Concussion Quick Check	American Academy of Neurology	Google Play and Apple App Store	No	No	Yes	No	No	No	No	Yes	No	No
Concussion Info	Programming is Fun	Google Play	No	No	No	No	No	No	No	No	No	No
How to Treat a Concussion	nermine_92	Google Play	No	No	No	No	No	No	No	No	No	No
PACE Concussion	PACE Concussion	Google Play	Yes	N/A^c^	N/A	N/A	N/A	N/A	N/A	N/A	N/A	N/A

^a^mTBI: mild traumatic brain injury.

^b^CBT: cognitive behavioral therapy.

^c^N/A: not applicable.

**Table 3:** Mobile Application Rating Scale scores for each of the 13 apps.

App names	Section A: engagement	Section B: functionality	Section C: aesthetics	Section D: information	App quality	Section E: subjective	Section F: app specific
Concussion Coach	3.8	4.3	4.7	4.6	4.4	5.0	4.3
World Rugby Concussion App	4.4	5.0	4.8	4.3	4.6	4.5	4.0
CDC Heads Up! Concussion and Helmet Safety App	3.8	5.0	5.0	4.3	4.5	4.0	3.8
Concussion Awareness	2.4	2.8	2.3	2.7	2.6	1.8	2.7
Concussion Ed	3.6	4.8	4.3	4.0	4.2	3.5	4.0
LifeArmor	3.4	4.0	3.7	4.0	3.8	3.0	3.0
CCMI Concussion Tracker	3.4	4.5	4.7	4.1	4.2	3.3	3.7
Concussion Smart	3.2	4.8	4.3	3.4	3.9	3.0	3.7
Concussion NI	2.8	4.0	4.7	4.0	3.9	3.0	3.7
Concussion Quick Check	2.6	3.8	3.0	3.6	3.3	2.0	3.0
Concussion Info	2.4	2.3	2.3	2.3	2.3	1.8	2.7
How to Treat a Concussion	2.6	3.8	3.3	2.3	3.0	1.5	2.7
PACE Concussion	N/A^a^	1.0	3.0	1.3	1.8	1.0	1.0

^a^N/A: not applicable.

## Discussion

**Figure 3:**
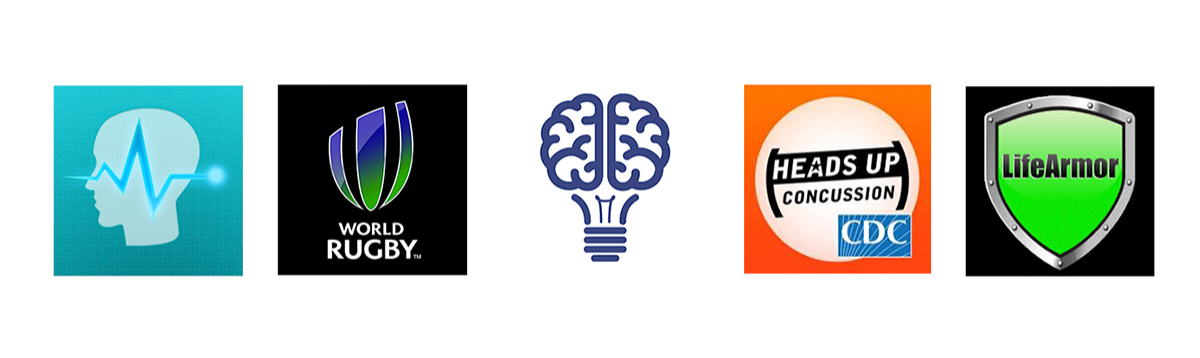
The top 5 scoring apps based on the Mobile Application Rating Scale (MARS) evaluation. Concussion Coach, World Rugby Concussion, Concussion Ed, CDC Heads Up!, and LifeArmor.

The decision of whether to use mHealth apps, as well as selecting which apps are appropriate, can be complex even without the addition of military health care, culture, and contexts. There are multiple considerations that need to be taken into account by both the person with an mTBI and/or a clinician using an app to assist with a psychoeducational intervention. To date, most of the available literature surrounding the use of mHealth and smartphone apps for mTBI focuses on diagnostic assessment or concussion recognition, and few studies have been published regarding app quality for psychoeducation and mTBI management. On the basis of the results of this search and subsequent MARS evaluations, 5 apps demonstrated superior app quality domains, including engagement, functionality, aesthetics, and information. The 5 highest-scoring apps included *Concussion Coach, World Rugby Concussion, CDC Heads Up!, Concussion Ed, and LifeArmor* (Tables 2 and 3; Figure 3). For the app-specific component of evidence-based merit (Information: Section D) for mTBI psychoeducation, the highest-scoring apps included *Concussion Coach*, *World Rugby Concussion*, *CDC Heads Up!*, *Concussion Ed*, and *LifeArmor* (Table 3; Figure 3).

Rating apps using the MARS highlighted features of the apps that may be beneficial to individuals with mTBI. Of the top apps, *World Rugby Concussion*, *CDC Heads Up!*, and *Concussion Ed* addressed acute mTBI recognition and management by providing parents, coaches, and individuals with instructions to follow in the event of sustaining an mTBI in a sporting context (ie, *remove from play*); these instructions are consistent with the best practices for sport-related mTBI [7,8]. All 5 apps provided a feature for symptom tracking. They also provided psychoeducational material for mTBI management and recovery, including information on mTBI acute management, recognition, and return to activity. *Concussion Coach* had the most robust psychoeducational resources of all the apps, with extensive information on sleep, headaches, goal setting, and cognition, in line with current best practice literature for mTBI and PCS psychoeducation for civilians and military populations [7,8,16,18]. Only 2 of the apps provided additional links to find community support in the United States (*Concussion Coach* and *LifeArmor*). Overall, the psychoeducational content provided was consistent with the current evidence base for mTBI recognition and management for both civilian and military populations. At no point was the information observed contraindicated for the recognition, management, and recovery of mTBI. The top-5 rated apps provided references or a link to a web resource where references could be found. References were evidence-based materials and seminal publications on mTBI management and best practices.

Most app users will be familiar with the 5-star rating system of the Google Play Store and the Apple App Store as a subjective manner for users to provide feedback on apps. Within this star rating system, the scores of the aforementioned top apps, as rated by the MARS, varied greatly. In the Apple App Store, *Concussion Coach, World Rugby Concussion*, and *Concussion* *Ed* each had 5 out of 5 stars, with *CDC Heads Up!* and *LifeArmor* not having enough ratings to formulate an average score. In the Google Play Store, *CDC Heads Up!* had 5 out of 5 stars, whereas *World Rugby Concussion* had 4.5 stars, *Concussion Coach* and *LifeArmor* had 4 stars, and *Concussion Ed* had 3 stars. It was noted that many of these scores were deemed as having less than 10 reviews. Although the 5-star rating system of the platforms could be helpful for some users deciding on whether to use the app, it is clear that the MARS provides greater overall reliability and validity because of being less subjective and having defined scoring criteria.

Although scoring from the MARS was helpful in assessing these health apps and providing a numeric score and ranking, there are many additional factors that need to be considered. These factors will be further discussed, including app platform, developer, internet requirement, cost, frequency of updates, language, additional features, acknowledgment of mental health, accessibility, and military specificity.

### App Platform

On searching both the Apple App Store and the Google Play Store, it was evident that the Google Play Store contained more apps; however, the Apple App Store produced a more refined and relevant search results (Figure 1). The requirements to publish apps vary between the Apple App Store and the Google Play Store, which influence the quality and volume of apps available. The Apple App Store has more rigorous review processes before allowing an app to be available to the public, which results in a more restrictive selection [32]. In addition, apps that score poorly in their customer five-star rating system risk being removed from the platform [32]. The Apple App Store may be a better option for finding adequate and usable apps for psychoeducation as a person with active symptoms of mTBI may struggle with the volume of results and the need to filter those that are not relevant [29]. Availability in both the Google Play Store and the Apple App Store will maximize the availability of the app to the target audience. It is evident that a clear title and description is key for finding and using an app [29].

### Developer

Ideally, the developer of the app is an organization whose policies and protocols are based on evidence-based, peer-reviewed literature, such as a government or an academic institution. The most common evidence-based apps are those that were developed through nationally competitive government or research funding and have undergone rigorous research ideally with randomized control trials [2]. None of the results yielded apps that had been researched to this degree. Additionally, health care app developers do not always involve medical professionals in app development; therefore, apps may have limited referencing and make misleading claims [33]. When health providers recommend apps, they need to be competent in the evidence-based literature on the topic to evaluate the apps for the quality of the content and ensure that it is in line with the desired therapeutic interventions and outcomes [3]. Although this expectation may be realistic for health care professionals who are clinically trained in a military health care system, it could be an unattainable expectation for military members in distress from an mTBI who are exploring mobile resources for themselves [4].

### Internet Requirement and Log-In

Most of the apps reviewed did not require an internet connection to function once downloaded and installed. This may be an asset for those apps intended for the military population as access to the internet may not always be available, especially when on deployment, in rural areas, or on a military exercise. Additionally, many smartphone users have limited data and are reliant on Wi-Fi. The requirement of a log-in and an account may benefit users in that they can save more of their customizations and information within the app and virtually share their progress with health care professionals. Regular access to the internet, however, may be required as well as the sharing of more personal information within the app. The top-rated apps, *Concussion Coach, World Rugby Concussion, CDC Heads Up!, Concussion Ed, and LifeArmor* (Figure 3)*,* did not require internet access after the app was initially downloaded to the device. In addition, they did not require a log-in or an account that negated the need for additional personal data to be stored and transmitted.

### Cost

Cost is also a factor in selecting the right app for health care needs, and health care professionals recommending the use of apps should consider the benefits and drawbacks of free versus paid apps. Free apps are more accessible and more likely to be used but may come with intrusive advertisements that may be distracting and confusing for someone experiencing cognitive, vestibular, or visuospatial dysfunction from mTBI. Paid apps may have fewer or no advertisements but are less likely to be downloaded [34]. A 2015 study concluded that although 93% of smartphone users download apps, only 35.8% will purchase apps that have an associated cost [34]. All 13 apps in this study were free to download and did not provide an option to pay for an upgraded version of the app. Advertisements were either absent or subtle enough to not disrupt the user experience.

### Up-to-Date

Several apps were outdated and had not undergone a version update in several years. All apps encouraged periods of complete rest after an mTBI; however, the evidence base is evolving toward a more active approach to recovery [7,17]. Although no regulations or standards exist regarding the frequency with which a developer should update an app, these apps should ideally be updated regularly enough to reflect the rapidly changing evidence base and recommendations regarding psychoeducation and mTBI management [28]. The 5 top-rated apps had received updates within 24 months of the app search, except the French version of *Concussion Ed* (last updated version in 2017).

### Language

*Concussion Ed* was the only app available in both of Canada’s official languages, English and French. All other apps reviewed were only available in English. If the CAF wishes to use a standard app across all regions, there is currently only 1 available option that will meet the official language needs of the country.

### Return to Activity

Of the 13 apps reviewed, 6 contained education specific to return to activities, such as sports, work, military duty, and/or school. Of the top 5 rated apps, the *World Rugby Concussion, CDC Heads Up!,* and *Concussion Ed* included this component and had concrete, specific examples for activity grading of frequency, volume, and intensity for both physical and cognitive tasks. The suggested return to play, school, or work guidance was consistent with evidence-based and best practice recommendations [7,8]. Guidance on return to activity is a common reason civilians and military members alike seek guidance from health care professionals [17]. This is also an area that is rapidly evolving in the literature as recent studies increasingly promote a more active recovery in contrast to previous recommendations that advocated for a longer period of complete rest immediately after sustaining an mTBI [7,8,17].

### Symptom Tracking

Of the 13 apps, 6 had a component of interaction with the app that allowed for customized day-to-day symptom tracking. Symptom tracking features addressed symptoms such as dizziness, headaches, balance issues, cognitive dysfunction, nausea, visual disturbances, sensitivity to noise, hearing difficulties, fatigue, sleep disturbances, and mood changes. Consistent with best practice recommendations, some apps utilized evidence-based outcome measures such as the Neurobehavioral Symptom Inventory to guide questions regarding symptom occurrence and severity [8,35]. Symptom tracking may have benefits such as allowing a patient with an mTBI to visualize improvement and better report their symptom status to their health care team. This may assist with decisions to re-engage with returning to certain activities. Conversely, frequent tracking could conceivably foster hypervigilance and preoccupation with symptoms, especially among anxious individuals [25].

### Acknowledgment of Mental Health

To provide evidence-based best practice care for mTBI, it is important that a holistic multidisciplinary approach is encouraged because of the variable experiences of those who experience mTBI [10]. This requires that mental health distress and diagnoses, such as PTSD, anxiety, and depression, are acknowledged as potential contributors to mTBI and PCS, especially in the military context where evidence of these comorbidities is strong [15,17-20]. The majority of apps reviewed did not contain reference to mental health. Although mental health symptomology and its comorbid relationship with mTBI is a key piece of the psychoeducational strategies recommended for mTBI management and intervention, only 1 app, *Concussion Coach,* explicitly acknowledged the comorbidity of mTBI and mental health conditions [8,15]. Furthermore, only 2 apps, *Concussion Coach* and *LifeArmor*, provided resources for mindfulness, goal setting, and cognitive behavioral therapy, which have been identified as components of a psychoeducational or integrated behavioral health intervention for mTBI and comorbid mental health conditions [15].

### Diagnostic Assessment or Recognition

Of the 13 apps, 7 had a component of acute mTBI assessment or recognition. Several were based on well-known and validated diagnostic tools utilized in a health care context such as the Glasgow Coma Scale and the Sport Concussion Assessment Tool 3 or 5 (SCAT3/SCAT5) [7,36,37]. The SCAT3/SCAT5 is a commonly used and evidence-based mTBI screen recommended for use during sport [7,37]. Of the top 5 rated apps, 3 (*World Rugby Concussion*, *CDC Heads Up!*, and *Concussion Ed*) included this component. As all the apps in this review are indicated for use by the general public, the mTBI assessment and recognition could carry significant legal liability, which is a concern that has been voiced in other reviews [24,25]. As stated earlier, none of the available smartphone apps in Canada and the United States have been federally regulated or approved for use in diagnosing mTBI [25,26]. The diagnostic utility of apps in mTBI detection is a controversial topic with emerging evidence-based literature [38-40].

### Accessibility

Readability and function of a concussion app needs to be appropriate for an individual experiencing mTBI symptoms; however, most apps contain layouts and fonts that would be difficult to read and process for individuals with visuospatial or cognitive symptoms of mTBI. As symptoms of concussion may include light sensitivity, difficulty reading, and visual disturbances, larger fonts, high visibility text, pictures or diagrams, and an intuitive interface are very important for usability [41]. Only *Concussion Ed* had a customizable interface that allowed the user to easily enlarge the text. In the opinion of the authors, the apps that scored in the top 5 had the best visibility and usability of the 13 apps assessed. With mTBI and its symptomatology being multifaceted and complex, intervention and rehabilitation will vary widely depending on the needs of the person [20].

### Military Specificity

As mentioned earlier, the use of “military” as a search term confounded the app search with multiple games and other nonrelevant apps. It is unrealistic to expect someone to search through all the options, especially if they are experiencing distress and cognitive dysfunction, as may be the case with military members who sustained an mTBI or a PCS and/or have a mental health condition [42]. Given the rate at which new apps are entering the marketplace, the open access nature of app stores may impact the effectiveness of starting a search there; consequently, search results are not guaranteed to be consistent [42].

Only 2 apps, *Concussion Coach* and *LifeArmor*, provided military-specific resources. The majority of apps, as well as evidence-based literature on mTBI, focus on sport-related mTBI, which is common in the civilian population. Although military populations experience these types of mTBI, apps specific to military contexts would be an asset as there are multiple complexities within military organizations, cultures, occupational roles, and the environment to which civilians are not exposed [13].

Military members are exposed to a variety of physical and psychosocial variables, which either in isolation or in combination can exacerbate the severity, longevity, and dysfunctionality of mTBI symptoms [9-16,43]. Psychosocial factors that are prevalent at a higher rate in military populations include increased geographical isolation, alcohol consumption, mental health diagnoses (ie, depression, anxiety, and PTSD), chronic pain, TBI, and sleep disturbances, all of which can exacerbate mTBI symptoms [9-16]. In addition, military contexts necessitate higher levels of cognitive functioning. Cognitive dysfunction can potentially result in decreased efficiency and effectiveness, along with an increased risk of harm to self, the unit, and a mission [18]. Reduced physical, mental, and/or cognitive functioning may result in involuntary release from the CAF because of the inability to meet the Universality of Service criteria for employment, deployment, and fitness [44]. Moreover, as a CAF-SM transitions from the CAF to veteran status, PCS may continue to contribute to challenges within the transition processes, the family unit, civilian employment, leisure activities, and self-care [21].

Blast injuries are also more unique to military populations, with a portion of the mTBI sustained by military members during OEF and OIF being potentially attributable to members being in close proximity to explosions [5,9-13,43]. A blast mTBI is an injury to the brain leading to dysfunction that is the result of an explosion or a blast [13,43]. Despite differences among mechanisms of injury, no significant variations in mTBI symptoms and PCS caused by blast versus blunt force have been identified apart from a blast mTBI preceding more severe hearing loss [13]. Finally, repeated exposure to low-level head trauma, such as being in proximity to low-level blasts, use of a ram, or other weapon utilization, is another mechanism of possible head trauma that is unique to military and paramilitary personnel being explored in the literature [45]. More research is needed to determine whether these mechanisms cause differences in the presentation of mTBI and PCS, and whether military-specific mTBI needs to be addressed as a unique subset compared with civilian mTBI.

### Health Care Use and Future Direction

There is potential for psychoeducational apps for mTBI to be utilized in a clinical setting [29]. A health care provider could recommend a vetted app to assist with education, reassurance, and potentially behavioral change. This may reduce the need for multiple follow-up appointments and could assist with symptom reporting and monitoring [3]. Some apps have a feature that allows data to be electronically provided to the health care professional, which could reduce the administrative burden on both the health care professional and the patient as long as data sharing, privacy, and security are considered [26]. Although there could be some benefits, this area of mHealth remains novel and not without significant issues [1,27-29,33].

Currently, health-related mTBI apps, including those providing psychoeducation and included in this review, are not formally regulated by government agencies, although *Concussion Ed* is endorsed by Health Canada [46]. The purpose of such regulation would be to provide the consumer with confidence that the product can be safely used [26]. Until this is changed, the onus is on app developers and the app provider (ie, Apple or Google) to provide the consumer with a well-documented and described product and a clear indication as to the intended target group of the app [24]. The abundance of available health apps and the rapid rate at which they continue to be released indicate that it will take considerable time for agencies or regulatory bodies to monitor a virtual market for regulation [25,26].

None of the apps investigated were found to have rigorous peer-reviewed research published on the app-specific effectiveness of the psychoeducational or mTBI management components. The lack of empirical research to demonstrate effectiveness may be related to the short time frame during which mHealth apps have emerged, the speed at which their availability changes as well as the focus on diagnostic apps opposed to psychoeducational apps [25].

The type and volume of data gathered from an electronic device when an app is downloaded and the details of the electronically signed end user license vary [46]. App creation, app use, and data storage may all occur in different countries with different laws and regulations regarding data privacy and sharing of data collected through electronic means [47]. Data sharing and privacy is a consideration that requires attention from researchers, health care professionals, and the general public when deciding on which app to utilize or if app utilization is appropriate at all [33]. Future systems of app evaluation and research would benefit from adding a component that considers data sharing, storage, and privacy.

Technology acceptance and usability studies are also lacking for apps related to mTBI [24]. There are few early feasibility and compliance studies for mTBI apps published with small sample sizes that are not specific to the psychoeducational components of the apps [25]. Investigation into the feasibility, logistics, security, IT compatibility, and acceptance by health care providers and military members with mTBI is critical to the implementation of standardized practices in military health care systems. The rapid rate at which mHealth is evolving contrasts with the slower pace of traditional evidence-based research practices [25]. Exhaustive evaluation for effectiveness, efficacy, and usability through research may not be practical and new approaches to evidence may have to be considered [25]. New methods of research with novel tools, such as the MARS, may also need further consideration and acceptance to assist both with the rapid need for mHealth research and the regulation of health apps.

### Study Limitations and Strengths

This study has a number of limitations. First, the search and identification of apps was limited to 1 day, and given the fast-paced release of new apps, it may not have captured all apps available till date. Second, there are specific concerns regarding the use of the MARS as a rating tool for health apps. The MARS does not address data sharing, security, and privacy, which are important components to consider when making decisions regarding mHealth utilization. Furthermore, the MARS involves several potentially subjective responses by the raters, most notably in the area of app subjective quality. For instance, question 2 asks, “Is the app interesting to use? Does it use any strategies to increase engagement by presenting its content in an interesting way?” [2]. As such questions may be interpreted subjectively, mitigation strategies were implemented. These included rater participation in standardized video training before rating the apps, engagement of multiple raters in the rating process, and averaging ratings across raters [2,29].

The main strength of this study is the systematic, methodological evidence-based approach undertaken to evaluate the apps, including the use of an evidence-based tool and multiple rounds of elimination. In addition, the researchers engaged in this project have clinical experience, are employed in clinical settings, and routinely work with military and civilian populations who have sustained mTBI. They are also skilled at providing psychoeducational content to address mTBI and support subsequent recovery. The execution of the *a priori* process for app rating, coupled with the educational and clinical experience of the researchers, contributed to the validity and rigor of the app evaluation results and subsequent knowledge synthesis of findings. As mHealth is a rapidly evolving field, the brief time from app search and evaluation to manuscript preparation facilitated timely knowledge translation and integration into clinical practice.

### Conclusions

As a component of mHealth, smartphone apps have become widely available in recent years as app technology rapidly improves. Similar to civilians, military populations have also embraced the use of health apps, which may have advantages specific to the challenges and barriers faced by military personnel, including geographical isolation and stigma. Health apps have the potential to be an engaging and accessible means of providing psychoeducational information for mTBI management. Of the 13 apps reviewed in this study, 5 (*Concussion Coach, World Rugby Concussion, CDC Heads Up!, Concussion Ed,* and *LifeArmor)* were well-suited to provide evidence-based psychoeducational information on mTBI management. Only 2 (*Concussion Coach* and *LifeArmor*) contained information specific to military populations and addressed mental health information and strategies, which are a critical component of mTBI and PCS management and recovery [8]. Further research should investigate the applicability, technology acceptance, and usability specific to the utilization of psychoeducational apps among military members with mTBI at the patient and health professional level within military contexts such as garrison, training, and deployed environments.

## Abbreviations

CAF: Canadian Armed Forces
MARS: Mobile Application Rating Scale
mHealth: mobile health
mTBI: mild traumatic brain injury
OEF: Operation Enduring Freedom
OIF: Operation Iraqi Freedom
PCS: postconcussion symptom
PTSD: posttraumatic stress disorder
SCAT3: Sport Concussion Assessment Tool 3
SCAT5: Sport Concussion Assessment Tool 5
SM: service member
TBI: traumatic brain injury

## References

[1] Buijink AW, Visser BJ, Marshall L (2013). Medical apps for smartphones: lack of evidence undermines quality and safety. Evid Based Med.

[2] Stoyanov SR, Hides L, Kavanagh DJ, Zelenko O, Tjondronegoro D, Mani M (2015). Mobile app rating scale: a new tool for assessing the quality of health mobile apps. JMIR Mhealth Uhealth.

[3] Armstrong C, Edwards-Stewart A, Ciulla R, Bush N, Cooper D, Kinn J, Pruitt L, Skopp N, Blasko K, Hoyt T (2017). Department of Defense Mobile Health Practice Guide. Military Health System.

[4] O'Toole K, Brown C (2020). Evaluating the Quality of Resilience Apps for Military Members and Public Safety Personnel. J Mil Veteran Fam Health.

[5] Garber BG, Rusu C, Zamorski MA (2014). Deployment-related mild traumatic brain injury, mental health problems, and post-concussive symptoms in Canadian Armed Forces personnel. BMC Psychiatry.

[6] (2016). Health and Lifestyle Information Survey of Canadian Forces Personnel 2013/2014. Government of Canada.

[7] McCrory P, Meeuwisse W, Dvořák J, Aubry M, Bailes J, Broglio S, Cantu RC, Cassidy D, Echemendia RJ, Castellani RJ, Davis GA, Ellenbogen R, Emery C, Engebretsen L, Feddermann-Demont N, Giza CC, Guskiewicz KM, Herring S, Iverson GL, Johnston KM, Kissick J, Kutcher J, Leddy JJ, Maddocks D, Makdissi M, Manley GT, McCrea M, Meehan WP, Nagahiro S, Patricios J, Putukian M, Schneider KJ, Sills A, Tator CH, Turner M, Vos PE (2017). Consensus statement on concussion in sport-the 5 international conference on concussion in sport held in Berlin, October 2016. Br J Sports Med.

[8] Marshall S, Bayley M, McCullagh S, Berrigan L, Fischer L, Ouchterlony D, Rockwell C, Velikonja D (2018). Guidelines for Concussion /Mild Traumatic Brain Injury and Prolonged Symptoms. Ontario Neurotrauma Foundation.

[9] Armistead-Jehle P, Soble J, Cooper D, Belanger H (2017). Unique aspects of traumatic brain injury in military and veteran populations. Phys Med Rehabil Clin N Am.

[10] Schwab K, Terrio HP, Brenner LA, Pazdan RM, McMillan HP, MacDonald M, Hinds SR, Scher AI (2017). Epidemiology and prognosis of mild traumatic brain injury in returning soldiers: a cohort study. Neurology.

[11] Hoge CW, McGurk D, Thomas JL, Cox AL, Engel CC, Castro CA (2008). Mild traumatic brain injury in US soldiers returning from Iraq. N Engl J Med.

[12] Rona RJ, Jones M, Fear NT, Hull L, Murphy D, Machell L, Coker B, Iversen AC, Jones N, David AS, Greenberg N, Hotopf M, Wessely S (2012). Mild traumatic brain injury in UK military personnel returning from Afghanistan and Iraq: cohort and cross-sectional analyses. J Head Trauma Rehabil.

[13] Doneva SP (2018). Mild traumatic brain injury in military service personnel: key issues and considerations. J Mil Veteran Fam Health.

[14] Janak JC, Cooper DB, Bowles AO, Alamgir AH, Cooper SP, Gabriel KP, Pérez A, Orman JA (2017). Completion of multidisciplinary treatment for persistent postconcussive symptoms is associated with reduced symptom burden. J Head Trauma Rehabil.

[15] Cooper DB, Bunner AE, Kennedy JE, Balldin V, Tate DF, Eapen BC, Jaramillo CA (2015). Treatment of persistent post-concussive symptoms after mild traumatic brain injury: a systematic review of cognitive rehabilitation and behavioral health interventions in military service members and veterans. Brain Imaging Behav.

[16] Garber BG, Rusu C, Zamorski MA, Boulos D (2016). Occupational outcomes following mild traumatic brain injury in Canadian military personnel deployed in support of the mission in Afghanistan: a retrospective cohort study. BMJ Open.

[17] Quatman-Yates CC, Hunter-Giordano A, Shimamura KK, Landel R, Alsalaheen BA, Hanke TA, McCulloch KL, Altman RD, Beattie P, Berz KE, Bley B, Cecchini A, Dewitt J, Ferland A, Gagnon I, Gill-Body K, Kaplan S, Leddy JJ, McGrath S, Pagnotta GL, Reneker J, Schwertfeger J, Silverberg N (2020). Physical therapy evaluation and treatment after concussion/mild traumatic brain injury. J Orthop Sports Phys Ther.

[18] Besemann M, Godsell P, Mahoney N, Hawes R (2019). Traumatic Brain Injury in the Canadian Armed Forces: Epidemiology, Management, and Rehabilitation. Canadian Institute for Military and Veteran Health Research Forum.

[19] Radomski M, Davidson L, Voydetich D, Erickson M (2009). Occupational therapy for service members with mild traumatic brain injury. Am J Occup Ther.

[20] Radomski M, Weightman M, Davidson L (2019). Mild Traumatic Brain Injury Rehabilitation Toolkit. US Army MEDCoE.

[21] Jones C, Pike A, Brémault-Phillips S (2019). Brain Bootcamp: pre–post comparison findings of an integrated behavioural health intervention for military members with reduced executive cognitive functioning. J Mil Veteran Fam Health.

[22] Lewis TL, Boissaud-Cooke MA, Aungst TD, Eysenbach G (2014). Consensus on use of the term 'app' versus 'application' for reporting of mHealth research. J Med Internet Res.

[23] Tam-Seto L, Wood VM, Linden B, Stuart H (2018). A scoping review of mental health mobile apps for use by the military community. Mhealth.

[24] Lee H, Sullivan SJ, Schneiders AG, Ahmed OH, Balasundaram AP, Williams D, Meeuwisse WH, McCrory P (2015). Smartphone and tablet apps for concussion road warriors (team clinicians): a systematic review for practical users. Br J Sports Med.

[25] Kwan V, Bihelek N, Anderson V, Yeates K (2019). A review of smartphone applications for persons with traumatic brain injury: what is available and what is the evidence?. J Head Trauma Rehabil.

[26] (2019). The FDA Recommends Only Using Cleared or Approved Medical Devices to Help Assess or Diagnose a Head Injury, Including Concussion. FDA Safety Communication.

[27] O'Neill S, Brady RR (2012). Colorectal smartphone apps: opportunities and risks. Colorectal Dis.

[28] Huckvale K, Prieto JT, Tilney M, Benghozi P, Car J (2015). Unaddressed privacy risks in accredited health and wellness apps: a cross-sectional systematic assessment. BMC Med.

[29] Wyatt JC (2018). How can clinicians, specialty societies and others evaluate and improve the quality of apps for patient use?. BMC Med.

[30] Stec M, Arbour MW (2020). Wellness and disease self-management mobile health apps evaluated by the mobile application rating scale. Adv Fam Pract Nurs.

[31] Stoyanov S (2016). MARS Training Video. YouTube.

[32] (2020). App Store Review Guidelines. Apple Developer.

[33] Balebako R, Cranor L (2014). Improving app privacy: nudging app developers to protect user privacy. IEEE Secur Privacy.

[34] Abroms LC, Westmaas JL, Bontemps-Jones J, Ramani R, Mellerson J (2013). A content analysis of popular smartphone apps for smoking cessation. Am J Prev Med.

[35] Cicerone KD, Kalmar K (1995). Persistent postconcussion syndrome: the structure of subjective complaints after mild traumatic brain injury. J Head Trauma Rehab.

[36] Teasdale G, Jennett B (1974). Assessment of coma and impaired consciousness. A practical scale. Lancet.

[37] McCrory P, Meeuwisse W, Johnston K, Dvorak J, Aubry M, Molloy M, Cantu R (2009). Consensus statement on concussion in sport: the 3rd international conference on concussion in sport held in Zurich, November 2008. J Athl Train.

[38] Eghdam A, Bartfai A, Oldenburg C, Koch S (2016). How do persons with mild acquired cognitive impairment use information and communication technology and e-services? Results from a Swedish national survey. PLoS One.

[39] Fitzgerald S (2019). Not so fast, federal regulators warn. There is no approved device to diagnose concussion on the spot. Neurology.

[40] Kutcher JS, McCrory P, Davis G, Ptito A, Meeuwisse WH, Broglio SP (2013). What evidence exists for new strategies or technologies in the diagnosis of sports concussion and assessment of recovery?. Br J Sports Med.

[41] Crowle C (2010). Low Vision Rehabilitation: A Practical Guide for Occupational Therapists.

[42] Owings-Fonner N (2019). A Review of Meditation Apps for Adults. Let's Get Technical: A Review of the Latest Apps and Tools for Practicing Psychologists. American Psychological Assocaition.

[43] Bryden D, Tilghman J, Hinds S (2019). Blast-related traumatic brain injury: current concepts and research considerations. J Exp Neurosci.

[44] (2018). DAOD 5023-0, Universality of Service. Government of Canada.

[45] Rhea CK, Kuznetsov NA, Ross SE, Long B, Jakiela JT, Bailie JM, Yanagi MA, Haran FJ, Wright WG, Robins RK, Sargent PD, Duckworth JL (2017). Development of a portable tool for screening neuromotor sequelae from repetitive low-level blast exposure. Mil Med.

[46] (2019). Concussion Management Resources. Government of Canada.

[47] (2017). Understanding Mobile Apps. Federal Trade Commission.

